# Social information can potentiate understanding despite inhibiting cognitive effort

**DOI:** 10.1038/s41598-018-28306-z

**Published:** 2018-07-02

**Authors:** Maxime Derex, Robert Boyd

**Affiliations:** 1 Human Behaviour and Cultural Evolution Group, Department of Biosciences, University of Exeter, Penryn, TR10 9FE United Kingdom; 2 Laboratory for Experimental Anthropology – ETHICS (EA 7446), Catholic University of Lille, 59016 Lille, France; 3 Institute of Human Origins, Arizona State University, Tempe, AZ 85287 USA; 4 School of Human Evolution and Social Change, Arizona State University, Tempe, AZ 85287 USA

## Abstract

Both reasoning ability and social learning play a crucial role in human adaptation. Cognitive abilities like enhanced reasoning skills have combined with cumulative cultural adaptation to allow our species to dominate the world like no other. Thus, understanding how social learning interacts with individual reasoning ability is crucial for unravelling our evolutionary history. Here we describe a laboratory experiment designed to investigate the effect of social learning on individuals’ ability to infer a general rule about unfamiliar problems. In this experiment, social information had both positive and negative effects on individuals’ likelihood of inferring the rule. Social learners required more evidence to infer the rule than did individual learners, suggesting that social learning inhibits cognitive effort but social learning provided individuals with information that individual learners were unlikely to gather on their own, especially as the task became more difficult. When individuals are unlikely to discover useful information by themselves, social learning can potentiate understanding even though it reduces individual cognitive effort.

## Introduction

As compared to other animals, humans exhibit both a remarkable ability to learn from others^1–3^ and an unparalleled capability to reason about their environments^4,5^. The combination of these capacities contributed to our species’ ecological success^6–8^. Thus, understanding how social learning interacts with individual reasoning ability is crucial for unravelling our evolutionary history. Although evolutionary anthropologists have debated the relationship between social learning and reasoning ability^5,8,9^, they have not investigated the question experimentally.

From an evolutionary perspective, there are good reasons to think that the availability of social information will negatively affect individuals’ ability to make useful inferences about their environment. Social learning is adaptive because it allows individuals to acquire useful information from others without paying the cost of producing it themselves^10^ and because it allows individuals to learn selectively^11^. Knowledge that can be generalized to new situations can be useful but to acquire it learners must process observed information, generate hypotheses and test them against data. These costs can be avoided by copying others’ behaviours without bothering with explanatory generalization. Evidence from human and non-human experimental studies suggest that social learning reduces effort exerted by learners. For example, using a series of simple problems that elicited false associative intuitions, an experiment showed that the propagation of correct, socially acquired responses did not promote analytic reasoning in learners^12^. In non-human animals, various social foraging experiments showed that individuals that are allowed to scrounge on conspecifics’ food finding are less likely to learn food-related cues than asocial learners^13–15^.

However, there are also good reasons to predict that social information can promote individuals’ understanding. It has been argued that the only difference between social and asocial learning is where the information comes from^16,17^. In social groups, the availability of cheap social information may promote explanatory generalization because it provides access to additional information that helps social learners’ to extract general rules and principles about their environment. Cognitive psychologists have shown that people who solve problems in groups will be better able to deal with subsequent problems as individuals than people without previous group problem-solving experience. These experiments, however, typically allow group members to interact verbally (refs^18,19^ but see^20^). Verbal interactions and argumentation can certainly help individuals in groups to better understand their environment. However, both cultural transmission and evolution also occur in absence of verbal communication. Lab experiments, for example, show that individuals get better at producing spaghetti towers or virtual fishing nets when provided only with the output of other group members’ behaviours^21–23^. Furthermore, many forms of teaching in traditional populations do not rely on verbal interactions^24,25^. Thus, experiments in which individuals cannot interact verbally are needed in order to get a comprehensive account of the relationship between social learning and individual reasoning.

**Figure 1:**
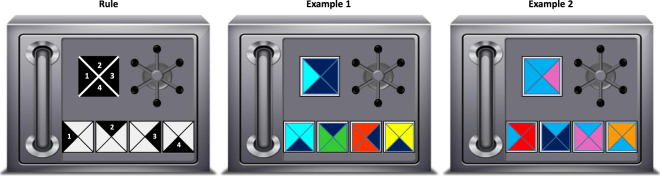
Game principle. The experimental task consisted in opening successive safe boxes using 4 keys that had to be dropped into 4 slots in any order (24 possible combinations). Each safe box displayed a single target composed from 4 triangles (black square on the left safe box). Safe boxes opened when players produced the unique combination that respected a simple rule unknown to them. In the Low Difficulty condition, the rule required individuals to drop the keys such as the left triangle on the first key (1) matched the left triangle on the target (1), the top triangle on the second key (2) matched the top triangle on the target (2), the right triangle on the third key (3) matched the right triangle on the target (3) and the bottom triangle on the fourth key (4) matched the bottom triangle on the target (4). Thus, the matching pattern went clockwise. Examples 1 and 2 display successful combinations. Note that keys’ colour and orientation are not consistent between combinations, yet they are both successful ones. Keys could not be rotated by participants. In the High Difficulty condition, the matching pattern did not go clockwise but was arbitrarily chosen (see Methods).

To investigate the effect of non-verbal social information on explanatory generalization, we developed an experiment in which participants were motivated to solve successive unfamiliar problems allowing for knowledge-transfer. The use of an unfamiliar task guaranteed that participants could not solve the task by referring to information they previously acquired outside the lab. The experimental task consisted in opening successive safe boxes using four keys that had to be dropped into four slots in any order (twenty-four possible combinations). Each safe box could be opened by producing the one combination that respected a rule unknown to the participants (Fig. 1). The production of successful combinations potentially allowed learners to infer this rule. Players had forty trials to open as many safe boxes as they could and were paid for the number of boxes opened. After each trial, they were told whether their combination was successful of not. If not, they were once again provided with the same safe box and the same keys. If they were successful, participants were provided with a new safe box and with new keys. At the end of the game, participants were asked to report the rule that produces successful combinations. Because the benefits of social learning are expected to be greater when information is costly to acquire individually^26^, we ran two different experimental conditions of this game: a *Low Difficulty* condition, with an easier-to-infer rule, and a *High Difficulty* condition, with a harder-to-infer rule (see Methods).

Participants were randomly assigned to one of the two treatments. They either played alone, the *individual* learning treatment, or in groups of four, the *social* learning treatment. Participants in the individual learning treatment had no access to social information over 40 trials. Participants in the social learning treatment potentially benefited from social information during the first 30 trials (the *learning* phase). After the 30^th^ trial, potential access to social information was removed so that social learners had to solve puzzles on their own for the last 10 trials (*test* phase). Social learners from the same group played simultaneously and were presented with safe boxes in the same order.

In this experiment, we wanted to measure the precise effect of non-verbal social information on individuals’ ability to infer general rules about their environment. To do so, we had to make sure that social learners were given no more feedback than individual learners across the experiment. Should that not be the case, individuals from the social learning treatment would be able to dismiss hypothesized rules more easily than individual learners^19^. For that reason, social information was limited to other group members’ successful solutions (i.e. other group members’ unsuccessful solutions were never displayed). Social learners were provided with social information when they didn’t open a specific safe box while another member of their group did. For example, if a participant solved safe box 1 at trial 3, unsuccessful members of the same group were automatically provided with the solution at trial 4 (alongside with the corresponding unopened safe box, see Supplementary Table 2). This protocol allowed us to make sure that social learners were given no more feedback than individual learners because the use of social information (i.e. reproducing a successful combination) did not provide any additional information to social learners: after solving a safe box using social information, participants were told that the combination they produced was successful (which they already knew). This means that both individual and social learners got feedback in each of 40 trials. The only difference between treatments was that, during the learning phase, social learners sometimes got “feedback” before producing a combination.

## Results and Discussion

**Figure 2:**
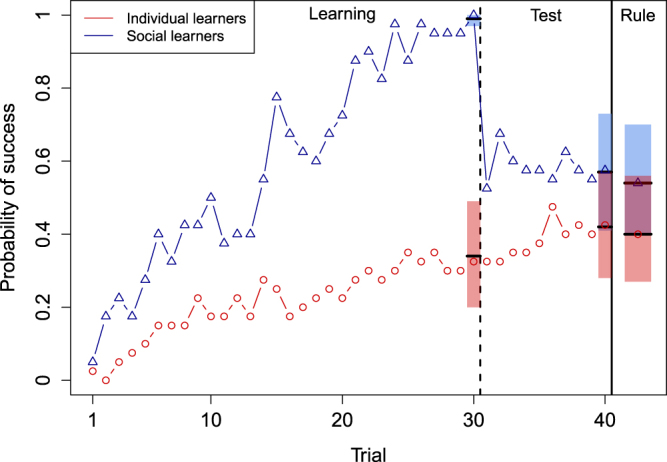
Individual probability of success across time in the Low Difficulty condition. The probability of success increased much faster in social learners (blue triangles, n = 40) than in individual learners (red diamonds, n = 40). Yet, the performance of social learners drastically dropped once they got isolated from their other group members (vertical dashed line). The probability of success at trial 40 and the probability of reporting the correct rule was slightly higher in social learners than in individual learners but did not differ statistically (see main text). Horizontal dashes show mean posterior probabilities and 95% HPDIs.

In the Low-Difficulty condition, social learners exhibited better performance than individual learners at the end of the learning phase (Social learning 95% Confidence Intervals: (3.37, 12.64), mean = 7.47). The mean posterior probability of opening the safe box at trial 30 was about 0.34 (95% Highest Posterior Density Interval: (0.20–0.49)) in individual learners and 0.99 in social learners (95% HPDI: (0.97–1)). Removing the access to social information in social learners resulted in a drop in performance (see Fig. 2). At trial 40, social learners were slightly more successful than individual learners but performance did not differ statistically between treatments (Social learning 95% CIs: (−0.21, 1.56), mean = 0.61). The mean posterior probability of opening the safe box at trial 40 was about 0.42 (95% HPDI: (0.28–0.57)) in individual learners and 0.57 in social learners (95% HPDI: (0.41–0.73)). At the end of the experiment, social learners were slightly more likely to report the correct rule than individual learners but again the probability of reporting the correct rule did not differ statistically between treatments (Social learning 95% CIs: (−0.32, 1.51), mean = 0.55). The mean posterior probability of reporting the correct rule at the end of the game was about 0.40 (95% HPDI: (0.27–0.56)) in individual learners and 0.54 in social learners (95% HPDI: (0.40–0.70)). These results suggest that social information primarily affects learners’ ability at producing successful solutions.

It is worth noting that with conjunctive rules, such as the one used in our experiment, a successful combination transmits more information about the rule than an unsuccessful combination^27^. Thus, the fact that social learners were not much more likely to infer the rule than individual learners despite being more successful during the test phase suggests that the exposure to social information decreased social learners’ cognitive effort.

**Figure 3:**
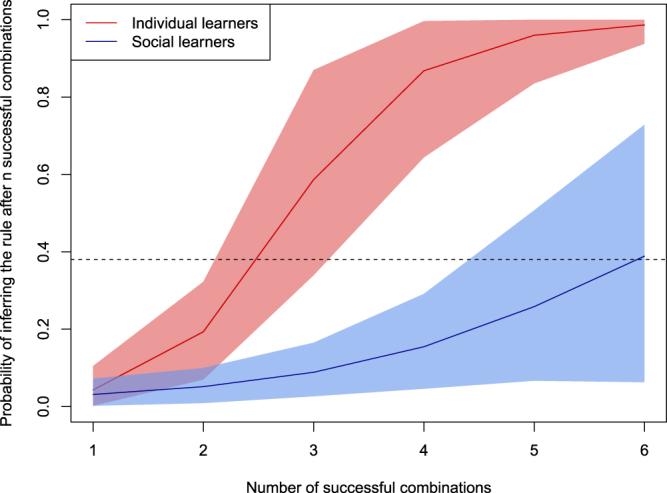
Individual probability of inferring the correct rule after *n* successful combinations. Individual learners (n = 34, red line) needed to produce less successful combinations to exhibit the same probability of inferring the rule than social learners (n = 39, blue line). Social learners’ data set were limited to periods in between the beginning of the experiment and the appearance of groups’ first rule guesser. During these periods, social learners were still actively searching for solutions and were provided with social information in an irregular fashion (see Supplementary Table 2). After the appearance of a first rule guesser, other group members were likely to be constantly provided with social information. Non-first guesser social learners (n = 29) produced between 10 to 27 successful combinations during the learning phase and reported the correct rule with a probability of 0.38 (dotted line). Our analyses suggest that constant access to social information further decreased individuals’ cognitive effort (see main text). Individual learners who never scored were removed from this analysis (6 out of 40). One social learner was removed because the rule he/she reported was considered as ambigous by the coders (see Supplementary Material for details). Solid lines show mean posterior probabilities and 95% HPDIs.

To test that assumption, we investigated the effect of each new successful combination on individuals’ probability of inferring the correct rule. For individual learners, we could identify precisely when individuals inferred the correct rule because once they did, they solved all subsequent problems (see Supplementary Table 1). Our analysis indicates that the probability of inferring the rule increased sharply after each successful attempt (Number of successful combinations 95% CIs: (0.78, 2.90), mean = 1.82). The mean posterior probability of inferring the correct rule after 2, 3 and 4 individually-produced successful combinations equalled to 0.19 (95% HPDI: (0.07, 0.32)), 0.59 (95% HPDI: (0.34, 0.87)) and 0.87 (95% HPDI: (0.64, 0.1)) respectively (see Fig. 3). These posterior probabilities strongly contrast with social learners’ performances. Social learners opened an average of 18 safe boxes (sd = 5.7) during the learning phase alone and yet only 51% reported the correct rule at the end of the game.

One might argue that social learners invested less in information processing because they had little incentive to try to infer the rule during the learning phase. Indeed, as soon as someone got the rule in a group of social learners (thereafter *first guesser*), he or she was likely to constantly provide the 3 other group members with solutions (see Supplementary Table 2). Repeated access to social information may thus have negatively affected their motivation to infer the rule. To control for the potential effect of being constantly exposed to social information on individuals’ motivation, we first analysed the relationship between the number of successful combinations and the probability of inferring the correct rule by social learners before the first guesser got the rule (i.e. in the early period of the learning phase, see Methods). By doing so, we limited our analysis to periods where individuals were provided with social information in an irregular fashion and were still actively searching for solutions. Results from this analysis show that additional pieces of information (i.e. successful combinations) had a smaller effect on the probability that social learners inferred the correct rule than the same information had on the probability that individual learners acquired the rule (Social Learning 95% CIs: (−1.70, 3.33), mean = 0.86; Social Learning x Number of successful combinations 95% CIs: (−2.27, −0.16), mean = −1.21). For social learners, the mean posterior probability of inferring the correct rule after having produced successful 2, 3 and 4 successful combinations equalled to 0.06 (95% HPDI: (0.01, 0.11)), 0.09 (95% HPDI: (0.03, 0.16)) and 0.16 (95% HPDI: (0.05, 0.28)) respectively (see Fig. 3). In a second analysis, we focused on the performance of non-first guessers (i.e. social learners who, at some point, were constantly provided with first guesser’s solutions). Non-first guessers produced between 10 to 27 successful combinations during the learning phase (mean = 17.6, s.d. = 5.8) and 38% of them reported the correct rule at the end of the experiment. Their probability of reporting the correct rule tended to increase with their success during the learning phase (Number of successful combinations 95% CIs: (−0.01, 0.28), mean = 0.13, see Supplementary Figure 1). The mean posterior probability of inferring the correct rule after being exposed to 10, 15 and 25 successful combinations equalled to 0.20 (95% HPDI: (0.03, 0.41)), 0.29 (95% HPDI: (0.11, 0.48)) and 0.58 (95% HPDI: (0.31, 0.84)) respectively. Thus, in order to exhibit a posterior probability of reporting the rule superior to 0.5, individual learners had to produce 3 successful combinations and social learners had to produce 7 when they were actively searching for solutions or 23 when they were constantly provided with solutions. These results suggest that (1) social learners were not processing pieces of information as extensively as individual learners as the former required more evidence than the latter to correctly infer the correct rule and (2) being constantly exposed to social information further decreases individuals’ cognitive effort.

An alternative explanation might be that, by constantly producing successful combinations, non-first guessers were less likely to infer the rule because they could not test their assumptions as efficiently as individuals who alternated between successful and unsuccessful combinations. For example, when data are consistent with different assumptions, negative examples can be of great help to disentangle potential solutions^28^. Our experimental task was however specifically designed to prevent different assumptions from being consistent with several successful combinations (see examples in Fig. 1). For that reason, social learners were able to reject wrong hypotheses even though they were only producing successful combinations. This is confirmed by our analysis about the performance of non-first guessers: non-first guessers who produced more successful combinations during the learning phase were more likely to report the correct rule at the end of the game.

**Figure 4:**
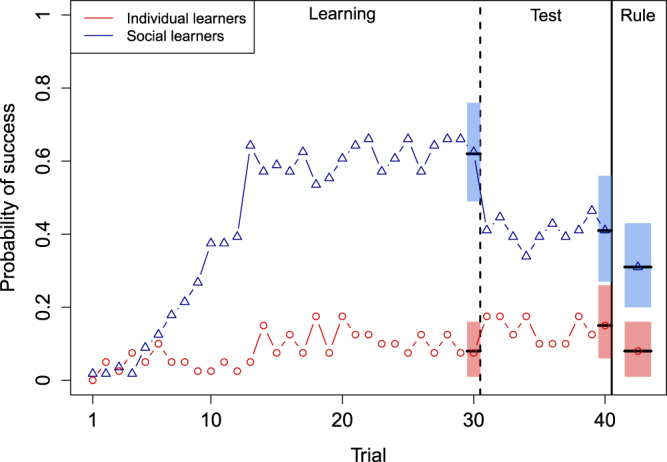
Individual probability of success across time in the High Difficulty condition. As in the Low Difficulty condition, the performance of social learners (blue triangles, n = 56) increased much faster than that one of individual learners (red diamonds, n = 40) before dropping after individuals’ isolation (vertical dashed line). However, in that condition, the probability of success and the probability of reporting the rule was higher in social learners than in individual learners. The performance of social learners plateaued because 6 out of 14 groups never inferred the rule in that treatment (see Supplementary Figure 3). Horizontal dashes show mean posterior probabilities and 95% HPDIs.

In the High Difficulty condition, the performance of social learners exhibited the same general trends. Their probability of success peaked during the learning phase before dropping during the test phase (Fig. 4). However, in this condition, social learners were more successful than individual learners at the end of both the learning phase (Social learning 95% CIs: (1.80, 4.47), mean = 3.10) and the test phase (Social learning 95% CIs: (0.42, 2.45), mean = 1.39). Social learners were also more likely to report the correct rule at the end of the game (Social learning 95% CIs: (0.54, 2.99), mean = 1.83). These results suggest that social information may potentiate the development of general knowledge when tasks get harder because when tasks are difficult, individuals are less likely to produce successful combinations on their own. Individual learners who did not produce any successful combinations were almost twice as frequent in the High Difficulty treatment (28% of them) than in the Low Difficulty treatment (15%). Most individual learners could not infer the rule because they did not encounter enough successful combinations. In comparison, social learners benefited from the lucky guesses generated by their other group members, which gave them a better chance of inferring the rule. For example, our results indicate that social learners who first got the rule in their group (whom we identified using streak of unaided successful combinations, n = 8, see Methods) always observed correct solutions by others before they were able to solve the problem on their own.

The small number of successful individual learners (3 out of 40 got the rule) in the High Difficulty condition prevented us from determining the effect of each new piece of information on the probability of getting the correct rule in the High Difficulty condition. Yet, as in the low difficulty treatment, social learners were exposed to many successful combinations (mean = 13, sd = 8.7) and displayed a relatively low probability of reporting the rule (0.33). This suggests that social learners exhibited low cognitive effort as observed in the low difficulty condition. If that is correct, our results suggest that the lower investment in information processing in social learners could be counter-balanced by their increased opportunity to be exposed to useful information. This is consistent with Henrich’s view of cultural products as promoter of greater understanding^29^. According to Henrich, efficient cultural solutions sometimes arise serendipitously and only then individuals speculate about why they work: “*by existing*, (…) *cultural products open a window on the world that facilitates the development of an improved causal understanding*”.

This experiment leads to several insights. First, it suggests that individuals flexibly invest in information processing depending on circumstances. This might reflect an adaptive strategy that allows social learners to exhibit appropriate behaviours while minimizing the cost of cognitive labour. This strategy would be in line with what cognitive scientists call *division of cognitive labour*^30^. Human populations have accumulated so much knowledge across time that no individual can produce all the information they rely on in their daily life. As a consequence, people outsource their understanding to informed individuals so that they do not rely only on their own knowledge but also on knowledge stored in others’ heads^31^. As shown in our experiment, this strategy doesn’t prevent efficient behaviours from spreading within populations. Actually, the fact that people don’t bother much about accurate explanation may positively affect cultural evolution. As pointed out before, cultural repertoires are full of causally opaque, sometimes even counter-intuitive, behaviours that are not easily understood by learners^29,32^. If people didn’t acquire behaviours unless they understood why they were efficient, successful behaviours that are not causally transparent could not persist^29^. By acquiring behaviours that are correlated with success/efficiency, even though they do not understand why these behaviours work, social learners help successful solutions to spread and increase other group members’ probability of being exposed to that behaviour. This increases behaviours’ probability of reaching out skilled individuals (i.e. that would understand why solutions work) as documented in our experiment’s High difficulty condition and can further promote cultural accumulation.

Further research will be necessary to confirm whether our results actually reflect an adaptive strategy. If correct, it may help explain why active teaching, a method that requires students to engage in meaningful activities and think about what they are doing, has proved to be more efficient than traditional lecturing, during which students passively receive information from the instructor^33^. Recent research on social learning in birds also suggests that social information may have a greater effect on learning when it is mediated by active search^13^. If recurrent and predictable access to social information lowers information processing, as our experiment suggests, optimal levels of learning should be reached by encouraging individual investigation on the basis of a few pieces of relevant social information.

## Methods

### Participants

A total of 176 Arizona State University students (88 women and 88 men) were randomly selected from a database managed by the Elinor Ostrom Multi-Method Lab at Arizona State University and recruited by email. The recruitment email indicated that the experimental task will require participants to produce combinations of variously coloured-items and so requested colour-blind recipients to not sign up. The subjects ranged in age from 18 to 26 years (mean 20 years, s.d. 1.40 years). Participants received $5 for participating and an additional amount ranging from $5 to $20 depending on their own performance.

### Ethical statement

The study was carried out in accordance with the ethical standards of the Declaration of Helsinki and the Belmont Report. Ethical approval was given by Arizona State University IRB (code: STUDY00003697). All participants provided written, informed consent before taking part in the experiment.

### Procedure

The experiment took place in a computer room at the Elinor Ostrom Multi-Method Lab at Arizona State University. For each session, a maximum of 20 participants (exclusively male or female) were recruited and randomly assigned to one condition of the experiment. Participants sat at physically separated and networked computers and were randomly assigned to a group or worked alone (treatment). Players did not know who belonged to their group and were instructed that communication and note taking were not allowed. Before starting the experiment, participants were requested to enter their age and sex and were asked whether they were colour-blind. Participants could read instructions on their screens. The game lasted 20 minutes, after which subjects received a reward according to their performance ($0.5 per successful combination).

### Game principle

The participants played a computer game (programmed in Object Pascal with Delphi 7) in which they were asked to open a maximum of safe boxes across 40 trials. At each trial, players were provided with a safe box and four different “keys”. Each safe box displayed a coloured symbol (thereafter “target”) and 4 empty slots in which participants had to drop the keys. Participants were given 30 seconds to drop the four keys in any order (24 possible combinations). If participants changed their mind before having dropped all four keys, they could click on a “reset” button to start all over again. After the 30 seconds elapsed, participants were told whether their combinations were successful or not. If not, participants were provided with the same safe box and associated keys during the next trial, otherwise they were provided with a new safe box and new keys. Safe boxes’ target and keys differed according to the way they were coloured. Targets, as well as keys, were squares composed of 4 triangles, one of which differed in its colour (thereafter “lone triangles”). Each target and key was composed of only 2 colours and all sets of target/associated keys were composed of 5 colours as a whole (Fig. 1). Across the game, sets of target/associated keys differed according to the colours involved and targets and associated keys differed according to the positions of lone triangles. Safe boxes got opened when participants dropped keys such as the sequence of keys respected a rule, initially unknown from players, that was stable across the game. Safe boxes were presented in the same order to all participants.

### Rule difficulty

Two difficulty conditions were implemented and players were randomly assigned to one of the two conditions. These conditions have been validated empirically using pilot studies only with individual learners and differed in how successful combinations were produced. In the Low-Difficulty condition, combinations were successful when keys were dropped such as the left triangle of the first key matched the colour of the left triangle of the target, the top triangle of the second key matched the colour of the top triangle of the target, the right triangle of the third key matched the colour of the right triangle of the target and the bottom triangle of the fourth key matched the colour of the bottom triangle of the target (Fig. 1). Thus, the position of the triangle seen in the key that had to match the target went clockwise (Left-Top-Right-Bottom). In the High-Difficulty condition, the matching pattern did not go clockwise: Combinations were successful when keys were dropped such as the top triangle of the first key matched the colour of the top triangle of the target, the right triangle of the second key matched the colour of the right triangle of the target, the left triangle of the third key matched the colour of the left triangle of the target and the bottom triangle of the fourth key matched the colour of the bottom triangle of the target (Top-Right-Left-Bottom). In both conditions, the rule was stable throughout the game. 80 and 96 players participated in the Low- and High-Difficulty condition respectively. All conditions involved an equal number of men of women.

### Treatment

Players were randomly assigned to one of the two treatments. Participants either played alone, the *individual* learning treatment (Low Difficulty: n = 40; High Difficulty: n = 40), or in groups of four, the *social* learning treatment (Low Difficulty: n = 40; High Difficulty: n = 56). When in groups, participants potentially benefited from social information during the first thirty trials (the *learning* phase) before being isolated without social information for the last ten trials (*test* phase). Social learners were provided with social information when they didn’t open a specific safe box while another member of their group did. For example, if a participant solved safe box 1 at trial 3, unsuccessful members of the same group were automatically provided with the solution at trial 4 (alongside with the corresponding unopened safe box, see Supplementary Table 2). Social learners were never provided with unsuccessful solutions from their other group members in order to prevent them from dismissing hypothesized rules more easily than individual learners. During the test phase, social learners receive no social information, the same conditions as individual learners. All treatments involved an equal number of men of women and social learners played in single-sex groups (in order to facilitate statistical analyses).

### Tutorial and pre-game information

Before starting, players had to complete a tutorial during which basic actions, such as dragging and dropping keys had to be completed. Players were informed that they “*might occasionally be provided with successful combinations from other players*” but were not aware about the existence of the learning or test phases. Players were told that a stable rule determined whether combinations were successful or not. Finally, participants were informed that their monetary a reward were going to depend on the number of safe boxes they opened.

### Explanation coding

Rules reported by participants were extracted from the database without any information about which treatment individuals participated in. Rules were independently coded by the two authors as a binary variable. Two reported rules were considered by the two coders as ambiguous so that data associated with the associated individuals were discarded from the analysis investigating the probability of getting the rule (see Supplementary Material for details). One individual took part in the Social Learning treatment/Low-Difficulty condition, the other one took part in the Social Learning treatment/High-Difficulty condition. Data points associated with these two individuals were included in the analyses investing individuals’ success at the trials 30 and 40. Importantly, the analyses of individuals’ success at trial 40 are consistent with the analyses of individuals’ success at reporting the rule indicating that the removing of two data points in the latter analyses (one per analysis per condition) did not affect our results.

### Statistical analyses

Models were fitted within a Bayesian framework^34^ with weakly informative priors. Data and full details about our models are available as supplementary materials.

To estimate the probability of success at trial 30 (i.e. end of the learning phase), trial 40 (i.e. end of the test phase) and the probability of reporting the correct rule, we fitted logistic regressions with “Success” as the outcome variable and “Social Learning” as the predictor variable (see Statistical model 1 in Supplementary Materials for details).

To estimate the relationship between the production of successful combinations and the probability of inferring the rule, we first identify which pattern of successes in individual learners perfectly predicted rule guessing. We found that individuals who generated streak of 3 successful combinations reported the correct rule with a probability of 1. We considered that individuals inferred the rule after the second success of their streak of successful combinations (see Supplementary Table 1). Data were then reorganized to investigate the effect of each new successful combinations on learners’ probability of inferring the rule. Individual learners who inferred the rule after n successful combinations were assigned n lines in the dataset. The variable “Rule” was set to 0 for successful combinations comprised between 1 to n − 1 and was set to 1 for successful combination n. Individual learners who scored n times but did not infer the rule were assigned n lines in the dataset, each line being paired with 0 for the variable “Rule” (see Supplementary Table 1 for details). Individual learners who never scored were removed from the analysis (6 out of 40). For each group of social learners, the data set was cut right after the trial during which the first guesser inferred the rule (see Supplementary Table 2). By doing so, we limited our analysis to periods where social learners were provided with social information in an irregular fashion and were still actively searching for solutions. The methodology explained above was then applied to social learners. First guessers who inferred the rule after n successful combinations were assigned n lines in the dataset. The variable “Rule” was set to 0 for successful combinations comprised between 1 to n-1 and was set to 1 for successful combination n. Non-first guessers who scored n times before the first guesser got the rule were assigned n lines in the dataset, each line being paired with 0 for the variable “Rule” (see supplementary table 2 for details). We fitted a mixed effects logistic regression with “Rule” as the outcome variable, “Success Number”, “Social Learning” and their interaction as predictor variables and “Player’s identity” as random effect (see Supplementary Tables 1 & 2 and Statistical model 2 for details).

To estimate the relationship between non-first guessers’ score during the learning phase and their probability of inferring the rule, we fitted a logistic regression with “Rule” as the outcome variable and “Score during the learning phase” as the predictor variable (see Supplementary Table 2.C and Statistical model 3 for details).

All analyses were conducted in R 3.2.1^35^ and models were fitted using map2stan, in the rethinking package^34^.

### Data Availability Statement

Raw data are available as supplementary materials.

## Electronic supplementary material

Supplementary Materials

Supplementary Dataset
