# Supplementary material for: Social information can potentiate understanding despite inhibiting cognitive effort
Source: Sci Rep. 2018 Jul 2;8:9980. doi: 10.1038/s41598-018-28306-z (PMC6028476; doi:10.1038/s41598-018-28306-z)
Supplement: Supplementary file 1 — Supplementary Materials [file 41598_2018_28306_MOESM1_ESM.pdf]

SUPPLEMENTARY MATERIALS

# Social information can potentiate understanding despite inhibiting cognitive effort

Maxime Derex[1,2,3] & Robert Boyd[3,4]

[1] Human Biological and Cultural Evolution Group, Department of Biosciences, University of Exeter, Penryn TR10 9FE, United Kingdom.

[2] Laboratory for Experimental Anthropology – ETHICS (EA 7446), Lille Catholic University, 59016 Lille, France.

[3] Institute of Human Origins, Arizona State University, Tempe, AZ 85287, USA.

[4] School of Human Evolution and Social Change, Arizona State University, Tempe, AZ 85287, USA.

A)

| | IL1 | | IL2 | |
|---|---|---|---|---|
| Trial | Safe box | Success | Safe box | Success |
| 1 | 1 | 0 | 1 | 0 |
| 2 | 1 | 0 | 1 | 1 |
| 3 | 1 | 1 | 2 | 0 |
| 4 | 2 | 0 | 2 | 0 |
| 5 | 2 | 0 | 2 | 1 |
| 6 | 2 | 0 | 3 | 0 |
| 7 | 2 | 1 | 3 | 0 |
| 8 | 3 | 1 | 3 | 0 |
| 9 | 4 | 1 | 3 | 0 |
| 10 | 5 | 1 | 3 | 0 |
| 11 | 6 | 1 | 3 | 0 |
| 12 | 7 | 1 | 3 | 0 |
| 13 | 8 | 1 | 3 | 0 |
| Rule | | 1 | | 0 |

B)

| Rule | Success number | Player |
|---|---|---|
| 0 | 1 | IL1 |
| 0 | 2 | IL1 |
| 1 | 3 | IL1 |
| 0 | 1 | IL2 |
| 0 | 2 | IL2 |

**Supplementary Table 1 | Datasets in the individual learning treatment.** In these illustrative examples, we consider that the experiment lasts 13 trials. A) Green cells illustrate successful trials. IL1 was successful at trial 3 and between trial 7 and trial 13. She reported the correct rule at the end of the experiment. IL1's streak of successful combinations indicates that she correctly inferred the rule by trial 8 (orange cell). IL2 was successful at trial 2 and 5 but did not report the correct rule at the end of the experiment. B) Data from A) were reorganized to investigate the effect of each new successful combination on individuals' probability of inferring the rule. IL1 did not infer the correct rule after having produced her two successful combinations but did so after the third one. IL2 did not infer the correct rule after having produced two successful combinations.

A)

| | | Group1 | | | | | | | | | | | |
|---|---|---|---|---|---|---|---|---|---|---|---|---|---|
| | | SL1 | | | SL2 | | | SL3 | | | SL4 | | |
| | Trial | Safe box | Success | SI | Safe box | Success | SI | Safe box | Success | SI | Safe box | Success | SI |
| Learning | 1 | 1 | 0 | 0 | 1 | 0 | 0 | 1 | 0 | 0 | 1 | 0 | 0 |
| | 2 | 1 | 0 | 0 | 1 | 0 | 0 | 1 | 1 | 0 | 1 | 0 | 0 |
| | 3 | 1 | 1 | 1 | 1 | 1 | 1 | 2 | 0 | 0 | 1 | 1 | 1 |
| | 4 | 2 | 0 | 0 | 2 | 0 | 0 | 2 | 0 | 0 | 2 | 0 | 0 |
| | 5 | 2 | 0 | 0 | 2 | 1 | 0 | 2 | 0 | 0 | 2 | 0 | 0 |
| | 6 | 2 | 1 | 1 | 3 | 0 | 0 | 2 | 1 | 1 | 2 | 1 | 1 |
| | 7 | 3 | 1 | 0 | 3 | 0 | 0 | 3 | 0 | 0 | 3 | 0 | 0 |
| | 8 | 4 | 1 | 0 | 3 | 1 | 1 | 3 | 1 | 1 | 3 | 1 | 1 |
| | 9 | 5 | 1 | 0 | 4 | 1 | 1 | 4 | 1 | 1 | 4 | 1 | 1 |
| | 10 | 6 | 1 | 0 | 5 | 1 | 1 | 5 | 1 | 1 | 5 | 1 | 1 |
| Test | 11 | 7 | 1 | | 5 | 0 | | 6 | 1 | | 5 | 0 | |
| | 12 | 8 | 1 | | 5 | 0 | | 7 | 1 | | 5 | 0 | |
| | 13 | 9 | 1 | | 5 | 0 | | 8 | 1 | | 5 | 0 | |
| | Rule | | 1 | | | 0 | | | 1 | | | 0 | |

B)

| Rule | Success number | Player |
|---|---|---|
| 0 | 1 | SL1 |
| 0 | 2 | SL1 |
| 1 | 3 | SL1 |
| 0 | 1 | SL2 |
| 0 | 2 | SL2 |
| 0 | 1 | SL3 |
| 0 | 2 | SL3 |
| 0 | 1 | SL4 |
| 0 | 2 | SL4 |

C)

| Rule | Score | Player |
|---|---|---|
| 0 | 5 | SL2 |
| 1 | 5 | SL3 |
| 0 | 5 | SL4 |

**Supplementary Table 2 | Datasets in the social learning treatment.** In these illustrative examples, we consider that the learning and test phases last 10 and 3 trials, respectively. The performance of a single group is shown. A) Green cells with red characters illustrate successful trials without social information. Green cells with black characters illustrate successful trials with social information. SL3 opened safe box 1 at trial 2 without social information. At trial 3, SL3 was provided with a new safe box while SL1, SL2 and SL4 were provided again with safe box 1 and the associated solution. SL1, SL2 and SL4 succeeded at trial 3. Individuals were provided with social information in an irregular fashion until SL1 (i.e. the first guesser) inferred the rule (orange cell). The period of active search ends after trial 7 (dashed line). From trial 8, SL1, SL2 and SL4 (non-first guessers) were constantly provided with social information up to the end of the learning phase. Once social information is removed, SL2 and SL4 stopped being successful and failed at reporting the correct rule. SL3 kept being successful after removing social information but we cannot tell when she inferred the rule. B) Data from the period of active search were reorganized to investigate the effect of each new successful combinations on individuals' probability of inferring the rule. SL3 inferred the correct rule after having produced her third successful combinations. SL1, SL2 and SL4 did not infer the rule at that time despite having produced two successful combinations. C) Non-first guessers scored 5 times during the learning phase but only SL 3 reported the correct rule at the end of the game.

**Statistical models:**

*Statistical model 1*:

This model was used to investigate the effect of social learning on individuals' success at trial 30 (the same model has been used to investigate individuals' success at trial 40 and individuals' success at reporting the correct rule).

$$S_i \sim \text{Binomial}(1, p_i)$$
$$\text{logit}(p_i) = \alpha + \beta_{SL}.SL_i$$
$$\alpha \sim \text{Normal}(0, 5)$$
$$\beta_{SL} \sim \text{Normal}(0, 5)$$

*S* indicates SUCCESS, *SL* indicates SOCIAL_LEARNING.

*Statistical model 2:*

This model was used to investigate the effect of each new successful combination on individuals' probability of inferring the rule in both treatments. In this analysis, the dataset was limited to periods of active search for social learners (see above).

$$R_i \sim \text{Binomial}(1, p_i)$$
$$\text{logit}(p_i) = \alpha + \alpha_{\text{PLAYER}[i]} + \beta_N.N_i + (\beta_{SL} + (\beta_{NSL}.N_i)).\ SL_i$$
$$\alpha_{\text{PLAYER}} \sim \text{Normal}(0, \sigma_{\text{PLAYER}})$$
$$\alpha \sim \text{Normal}(0, 5)$$
$$\beta_N \sim \text{Normal}(0, 5)$$
$$\beta_{SL} \sim \text{Normal}(0, 5)$$
$$\beta_{NSL} \sim \text{Normal}(0, 5)$$
$$\sigma_{\text{PLAYER}} \sim \text{HalfCauchy}(0, 2)$$

*R* indicates RULE, *N* indicates SUCCESS_NUMBER, *SL* indicates SOCIAL_LEARNING.

*Statistical model 3:*

This model was used to investigate the effect of individuals' score during the learning phase on their probability of inferring the rule. In this analysis, the dataset was limited to non-first guesser social learners (see above).

$$R_i \sim \text{Binomial}(1, p_i)$$
$$\text{logit}(p_i) = \alpha + \beta_S.S_i$$
$$\alpha \sim \text{Normal}(0, 5)$$
$$\beta_S \sim \text{Normal}(0, 5)$$

*R* indicates RULE, *S* indicates SCORE.

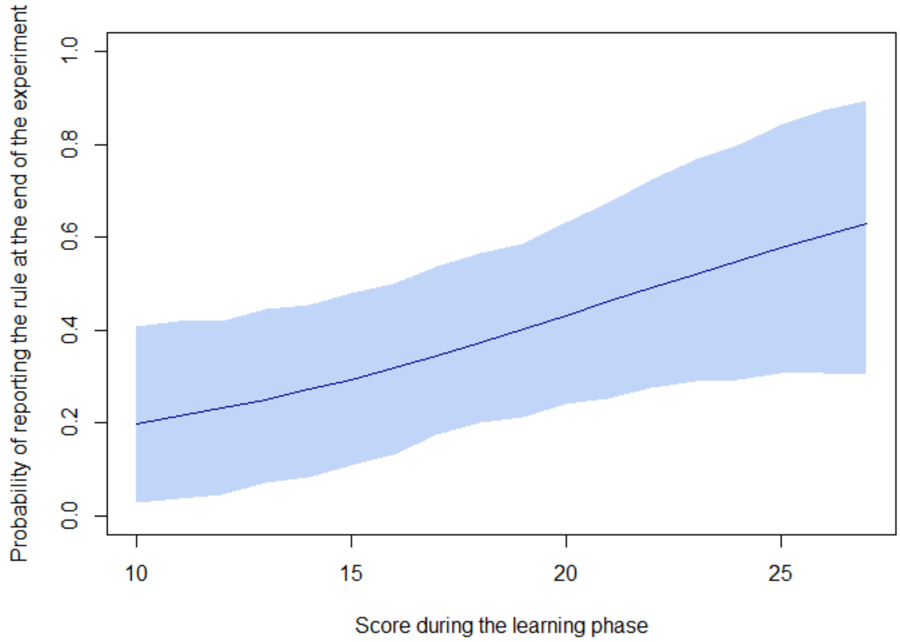


**Supplementary Figure 1 | Non-first guesser social learners' probability of reporting the correct rule according to their score during the learning phase.** Non-first guessers (n = 29) produced between 10 to 27 successful combinations during the learning phase (mean = 17.6, s.d. = 5.8). Individuals who produced more successful combinations tended to be more likely to report the correct rule (Number of successful combinations 95% CIs: (-0.01, 0.28), mean = 0.13).

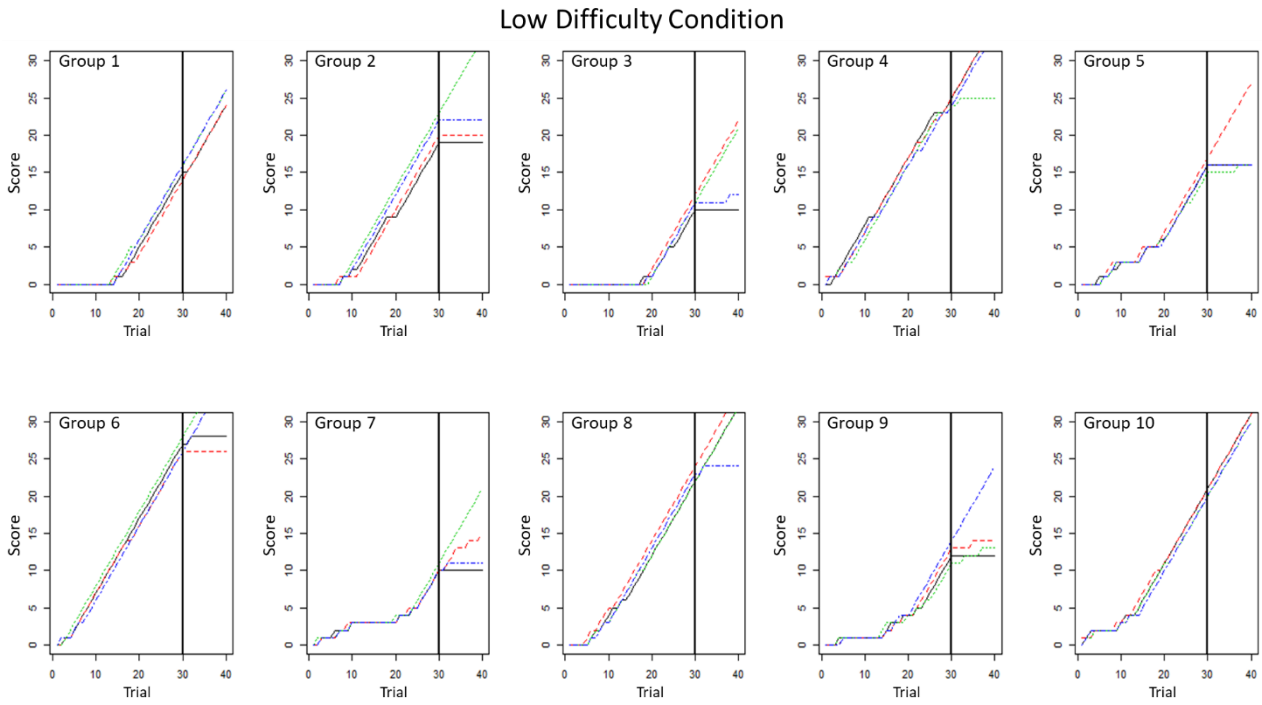


**Supplementary Figure 2 | Social learners' score across time in the Low Difficulty condition.** Each plot shows the score of 4 social learners of the same group. The appearance of groups' first rule guesser drove the performance of the other group members during the learning phase. The vertical line shows the end of the learning phase.

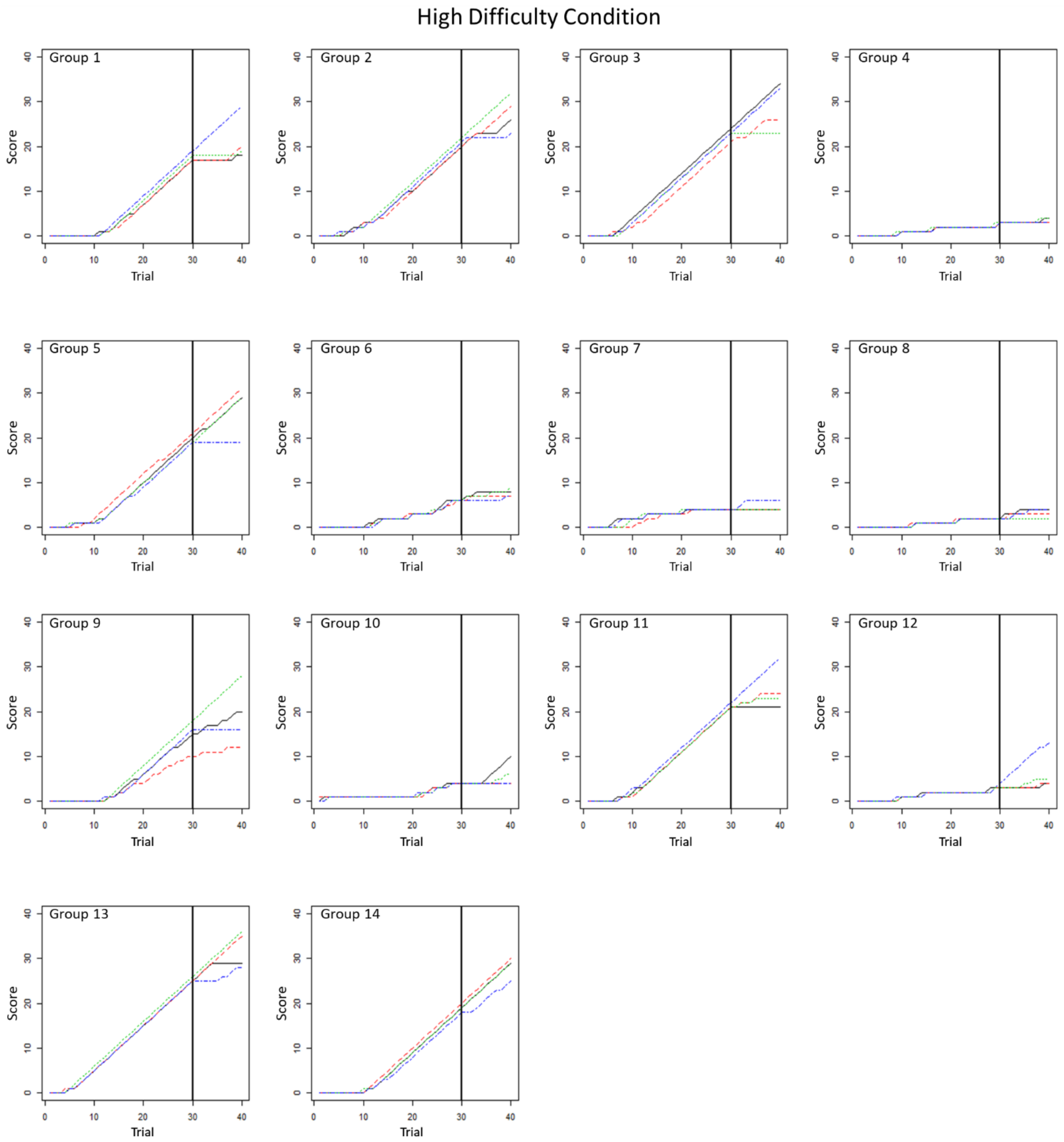


**Supplementary Figure 3 | Social learners' score across time in the High Difficulty condition.** Each plot shows the score of 4 social learners of the same group. The appearance of groups' first rule guesser drove the performance of the other group members during the learning phase. The vertical line shows the end of the learning phase. 8 out of 14 groups inferred the rule in that treatment.

# Examples of rules given by the participants:

## Low difficulty:

### Rules considered as correct:

1- *“The order of the colors had to match the key starting from the left triangle and going clockwise.”*
2- *“The color of the left triangle in had to match the main square first, then the top triangle, then the right triangle, and finally the bottom triangle in that order.”*
3- *“The first box correlated to the triangle on the left, the second box to the top triangle, the third box to the right triangle, and the last box to the bottom triangle.”*

### Rules considered as incorrect:

1- *“I thought it was a mathematical problem where you add the "odd" color in certain places, and subtract it in others.”*
2- *“Arranging the tiles by chromatic order.”*
3- *“I have no idea.”*

### Rule considered as ambiguous (n=1):

1- *“If the left quarter was different, then the first block would be the one with the majority that color, rotating clockwise in accordance with spots. Unless color said otherwise, first block pointed left and last pointed up.”*

## High difficulty:

### Rules considered as correct:

1- *“The rule was that it would take one triangle from each square. It started on the top and then added on the right, then the left then the bottom.”*
2- *“The rule was to first match up the color on the top, then the right, then the left, then the bottom.”*
3- *“First key determined top, second key determined right, third determined bottom, fourth key determined left.”*

### Rules considered as incorrect:

1- *“There was no successful combination.”*
2- *“The sides had to face triangles of the same color away from each other.”*
3- *“Cancelling out colors to make the combination and the order.”*

### Rule considered as ambiguous (n=1):

1- *“The first square used the top color, the second square used the an inverse of the right hand square for its color, the third square used an inverse of the left hand color square for its color, and the final.”*
